# Exosomes in depression: mechanistic insights, diagnostic potential, and therapeutic opportunities

**DOI:** 10.1038/s41380-026-03622-3

**Published:** 2026-04-28

**Authors:** Fei Wang, Jian Yang, Gang Wang

**Affiliations:** Beijing Key Laboratory of Intelligent Drug Research and Development for Mental Disorders; National Clinical Research Center for Mental Disorders; National Center for Mental Disorders; Beijing Anding Hospital, Capital Medical University, Beijing, China

**Keywords:** Depression, Diagnostic markers, Molecular biology

## Abstract

Exosomes are increasingly recognized as critical mediators of intercellular communication within the central nervous system and along the brain–periphery axis, with emerging relevance to the pathophysiology of depression. These nanoscale extracellular vesicles transport bioactive cargo—including microRNAs (miRNAs), proteins, lipids, and metabolites—that can modulate neuroinflammatory signaling, synaptic remodeling, hypothalamic–pituitary–adrenal (HPA) axis dynamics, and gut–brain communication. Converging evidence from human and preclinical studies suggests that alterations in circulating and brain-derived exosomal cargo are associated with major depressive disorder (MDD) and secondary depression following neurological insults such as traumatic brain injury (TBI), implicating shared inflammatory and synaptic pathways alongside distinct etiological drivers. Circulating neuron-enriched exosomes carrying miRNAs such as miR-16 and miR-124 have been proposed as minimally invasive biomarkers reflecting brain-relevant molecular alterations. However, most human data remain cross-sectional and associative, with limited longitudinal replication and incomplete validation of cellular origin. Therapeutically, mesenchymal stem cell (MSC)–derived exosomes demonstrate antidepressant-like effects in rodent models through modulation of microglial activation, enhancement of synaptic plasticity, and attenuation of neuroinflammatory cascades. Engineered exosomes further offer a potential platform for targeted delivery of anti-inflammatory or neuroplasticity-enhancing cargo. Induced pluripotent stem cell (iPSC)-derived exosomes represent a potentially scalable and standardizable therapeutic platform. Despite this promise, substantial barriers impede clinical translation. Isolation workflows—including ultracentrifugation, size-exclusion chromatography, and immunoaffinity capture—vary in yield, purity, and reproducibility, complicating cross-cohort comparability. Moreover, definitive attribution of vesicle origin in peripheral biofluids remains technically constrained, and standardized reporting frameworks are inconsistently applied. Advances in multi-omics profiling, high-throughput sequencing, mass spectrometry, and spatially resolved molecular analyses may improve mechanistic resolution and biomarker robustness. Collectively, exosome research in depression resides at an early but rapidly evolving translational stage. Rigorous methodological standardization, longitudinal cohort validation, and mechanistic dissection across primary and injury-related depression will be essential to determine whether exosome-based diagnostics and therapeutics can fulfill their potential within precision psychiatry.

## Introduction

Major depressive disorder (MDD) is a highly prevalent and recurrent psychiatric condition and a leading contributor to global disability. According to the World Health Organization, depression ranks among the top causes of disease burden worldwide [1]. Despite extensive investigation, its pathophysiological basis remains incompletely resolved, limiting mechanistic stratification and precision therapeutic development.

Recent studies have identified multiple biological processes contributing to the pathogenesis of depression, including, but not limited to, the following: Neuroinflammation: Converging clinical and experimental evidence indicates that subsets of patients exhibit elevated peripheral and central inflammatory markers [2–6]. Increased circulating concentrations of interleukin-6 (IL-6), tumor necrosis factor (TNF), and interleukin-1β (IL-1β) have been reported in meta-analyses. These inflammatory signals are associated with microglial activation, altered blood-brain barrier (BBB) integrity, and synaptic remodeling [7, 8]. However, inflammatory signatures are heterogeneous and appear most prominent in specific clinical subtypes (e.g., treatment-resistant or high-inflammatory phenotypes), rather than universally across all individuals with MDD. Neurotransmitter dysregulation: The monoamine hypothesis historically posited that reduced serotonergic, noradrenergic, and dopaminergic signaling underlies depressive symptomatology [9, 10]. Although monoaminergic modulation remains central to pharmacotherapy, this framework is now understood to be incomplete. Glutamatergic and γ-aminobutyric acid (GABA) signaling, synaptic plasticity, and neurotrophic mechanisms—particularly those involving brain-derived neurotrophic factor (BDNF)—have emerged as critical components of contemporary models. Brain-gut axis alterations: The brain–gut axis comprises bidirectional communication among the central nervous system (CNS), autonomic nervous system, immune pathways, and the intestinal microbiota. Reduced microbial diversity and altered metabolite profiles have been described in MDD cohorts [11, 12]. These alterations may influence mood-relevant circuitry through immune modulation, tryptophan metabolism, and synaptic plasticity pathways. Causal directionality, however, remains incompletely established.

**Fig. 1:**
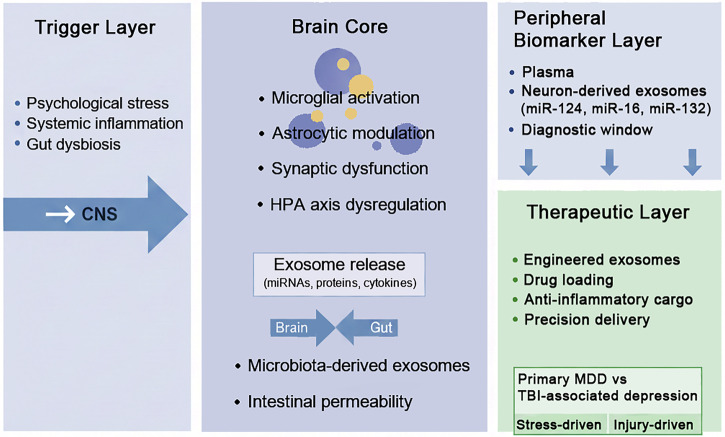
Schematic overview of exosome-mediated neuroimmune communication in primary and secondary depression. Psychological stress, systemic inflammation, and gut dysbiosis converge to modulate exosome biogenesis and cargo composition in both central and peripheral compartments. Within the central nervous system (CNS), neurons, astrocytes, and microglia release exosomes enriched in regulatory microRNAs (miRNA), inflammatory mediators, and synaptic proteins that influence neuroinflammation, synaptic remodeling, and hypothalamic-pituitary-adrenal (HPA) axis dynamics. Bidirectional communication between the brain and gut is mediated in part by circulating vesicles, which may traverse a functionally altered blood-brain barrier (BBB) and contribute to immune-neural crosstalk. Circulating neuron-enriched exosomes carrying candidate microRNAs (e.g., miR-124, miR-16, miR-132) provide a minimally invasive window into brain-relevant molecular alterations. Therapeutically, engineered or stem cell-derived exosomes are being explored as targeted delivery platforms for anti-inflammatory or neuroplasticity-enhancing cargo. Shared and divergent mechanisms between primary major depressive disorder (MDD) and traumatic brain injury (TBI)-associated depression are highlighted, illustrating convergent inflammatory and synaptic pathways alongside distinct etiological drivers (stress-related dysregulation versus injury-induced neurodegeneration). CNS central nervous system, BBB blood–brain barrier, HPA axis hypothalamic–pituitary–adrenal axis, miRNA microRNA, MDD major depressive disorder, TBI traumatic brain injury, IL-6 interleukin 6, TNF-α tumour necrosis factor alpha, BDNF brain-derived neurotrophic factor.

Within this evolving systems framework, exosomes—30–150 nm extracellular vesicles (EVs) generated through multivesicular body (MVB) pathways—have gained attention as potential mediators of intercellular communication. Exosomes transport diverse cargo, including proteins, lipids, messenger RNA, microRNAs (miRNAs), and other non-coding RNAs [13–15]. Because vesicular content reflects the physiological state of the originating cell, exosomes have been proposed as both mechanistic participants and peripheral readouts of CNS processes (Fig. 1). Preclinical and clinical studies suggest that exosomal signaling may intersect with neuroinflammatory pathways, synaptic remodeling, and gut-brain communication. Nonetheless, much of the current human literature remains cross-sectional and associative. Sample sizes are typically modest, diagnostic stratification is variable, and methodological heterogeneity in isolation and characterization complicates reproducibility.

This review provides a comprehensive synthesis of recent advances in the role of exosomes in depression, with particular emphasis on their involvement in core pathophysiological mechanisms and their emerging diagnostic and therapeutic potential. Importantly, while a growing body of literature implicates exosomal signaling in depressive phenotypes, much of the current evidence remains associative rather than demonstrably causal. The majority of human investigations are based on cross-sectional designs characterized by modest sample sizes, diagnostic heterogeneity, and variability in biofluid processing and isolation methodologies. Accordingly, we explicitly distinguish correlational observations from experimentally substantiated mechanistic findings and critically evaluate the strength, methodological rigor, and reproducibility of the existing evidence. By integrating current knowledge while transparently addressing conceptual and technical limitations, this review aims to refine the translational framework of exosome research in depression and delineate priorities for future investigation (Fig. 1).

## Fundamental properties of exosomes in the nervous system

Exosomes originate from inward budding of endosomal membranes, generating intraluminal vesicles within MVBs [16, 17]. Fusion of MVBs with the plasma membrane releases these vesicles into the extracellular space. Most cell types—including neurons, astrocytes, microglia, oligodendrocytes, immune cells, and stem cells—secrete exosomes [18–26]. Exosomes are enriched in characteristic surface markers such as tetraspanins (CD9, CD63, CD81), integrins, and major histocompatibility complex (MHC) molecules. Their cargo includes heat shock proteins, signaling proteins, metabolic enzymes, DNA fragments, mRNA, and regulatory non-coding RNAs such as miRNAs and long non-coding RNAs (lncRNAs).

Exosomes play a pivotal role in intercellular communication, regulating numerous physiological and pathological processes. They facilitate cell-to-cell signaling by transferring proteins and RNAs, modulate immune cell activity, and, under pathological conditions, contribute to disease propagation [13–15, 27–29]. In the nervous system, exosomes exhibit unique functions, contribute to modulation of synaptic function, regulation of neuroimmune signaling, intercellular metabolic coordination and propagation of pathological proteins in neurodegenerative conditions.

In neurotransmitter transmission, exosomes contain enzymes and regulatory RNAs associated with neurotransmitter metabolism and synaptic function [30–32]. Vesicular cargo has been reported to include glutamate decarboxylase (GAD), an enzyme involved in GABA synthesis, or monoamine oxidase (MAO), which participates in the metabolism of dopamine and serotonin (5-HT). By releasing these vesicles into the synaptic cleft or extracellular environment, exosomes regulate neurotransmitter concentrations and availability. Furthermore, they facilitate signal transmission between pre- and postsynaptic neurons, maintain synaptic plasticity, and modulate neuronal activity [33]. Additionally, exosomes may influence neurotransmitter efficacy by altering receptor distribution or sensitivity on the postsynaptic membrane [34]. Exosomal BDNF and TrkB-associated molecules have been implicated in activity-dependent plasticity. Altered exosomal levels of these components have been observed in stress paradigms and human depressive cohorts, although causality remains to be clarified.

The role of exosomes in neuroinflammation has garnered increasing attention. Exosomes participate in bidirectional regulation of neuroimmune processes. Microglia-derived vesicles may amplify inflammatory cascades through cargo enriched in cytokines, danger-associated molecular patterns, or pro-inflammatory miRNAs. Conversely, exosomes enriched in miR-124 or miR-146a have been shown in experimental systems to attenuate nuclear factor κB (NF-κB) signaling and reduce cytokine production (e.g., IL-1β, TNF-α) [35–37]. This activity not only alleviates neuroinflammation but also promotes neuronal survival and regeneration to some extent. However, under pathological conditions, such as depression or neurodegenerative diseases, exosomes can act as mediators of inflammatory propagation. In these contexts, exosomes often carry elevated levels of pro-inflammatory factors, oxidative stress molecules, or pathogenic proteins (e.g., amyloid precursor protein fragments). Whether such changes are drivers of pathology or epiphenomena reflecting upstream immune activation remains unresolved.

Importantly, the cellular origin of exosomes may significantly influence their specific roles in the nervous system. For example, exosomes secreted by astrocytes are more likely to carry molecules related to neurotransmitter reuptake or metabolism [38, 39], whereas exosomes derived from microglia are more involved in modulating neuroinflammation [40, 41]. This source specificity highlights that the function of exosomes is determined by the state of their parent cells and environmental conditions, enabling them to mediate bidirectional regulation between neurotransmitter transmission and neuroinflammation.

Exosomes can traverse or influence the BBB under specific physiological and pathological contexts. While BBB permeability alterations are reported in subsets of depressed patients and in stress models, the quantitative contribution of vesicle trafficking to central–peripheral immune crosstalk remains an area of active investigation.

Exosomes also play a crucial role in the pathology of neurodegenerative diseases, such as Alzheimer’s disease (AD) and Parkinson’s disease (PD). In AD, exosomes have been shown to carry β-amyloid (Aβ), amyloid precursor protein (APP), β-site APP cleaving enzyme 1 (BACE1). potentially contributing to protein aggregation dynamics [42–48]. In PD, exosomes transport α-synuclein (α-syn) species and may facilitate inter-neuronal spread [49–59]. This mechanism may explain the characteristic spread of PD pathology from midbrain dopaminergic neurons to other brain regions. Furthermore, exosomes in PD patients have been shown to contain oxidative stress-related molecules and pro-inflammatory factors, which may exacerbate neuronal damage and death [60–62].

Beyond AD and PD, exosomes exhibit similar pathological roles in other neurodegenerative diseases, such as Huntington’s disease (HD) and amyotrophic lateral sclerosis (ALS). In HD, exosomes may carry mutant huntingtin protein (mHTT), facilitating its spread in brain tissues [63–65]. In ALS, exosomes can transport abnormally folded TAR DNA-binding protein 43 (TDP-43) or Superoxide dismutase 1 (SOD1) proteins, accelerating neuronal dysfunction [66–69]. These findings underscore the capacity of exosomes to redistribute bioactive—and potentially pathogenic—molecules across neural networks. However, extrapolation to psychiatric disorders requires caution, as depression lacks a single defining proteinopathy and is characterized by multifactorial systems dysregulation rather than discrete aggregate spread.

Collectivelly, exosomes represent context-dependent mediators of intercellular communication in the nervous system. Under physiological conditions, they contribute to synaptic homeostasis, neuroimmune regulation, and metabolic coordination across neuronal and glial networks. Under pathological states, however, vesicle-mediated transfer may facilitate the redistribution of inflammatory mediators, stress-associated molecular signals, or misfolded proteins, potentially amplifying circuit-level dysfunction. Rather than being inherently protective or deleterious, exosomes appear to reflect and propagate the biological state of their parent cells within a given microenvironment. While propagation of pathogenic proteins is a defining feature of several neurodegenerative disorders, depression is characterized by systems-level dysregulation without a singular proteinopathy, necessitating caution when extrapolating mechanistic models. Clarifying vesicle origin, cargo specificity, and causal contribution will be essential to determine whether exosomes serve primarily as biomarkers, mechanistic intermediaries, or therapeutic targets in psychiatric disease.

## Exosomes in the pathogenesis of depression

MDD is increasingly conceptualized as a disorder of systems-level dysregulation involving neuroinflammatory activation, altered neurotransmission, stress-axis imbalance, and disrupted brain–periphery communication. Within this framework, exosomes may function as dynamic intermediaries linking cellular stress responses to circuit-level dysfunction.

**Fig. 2:**
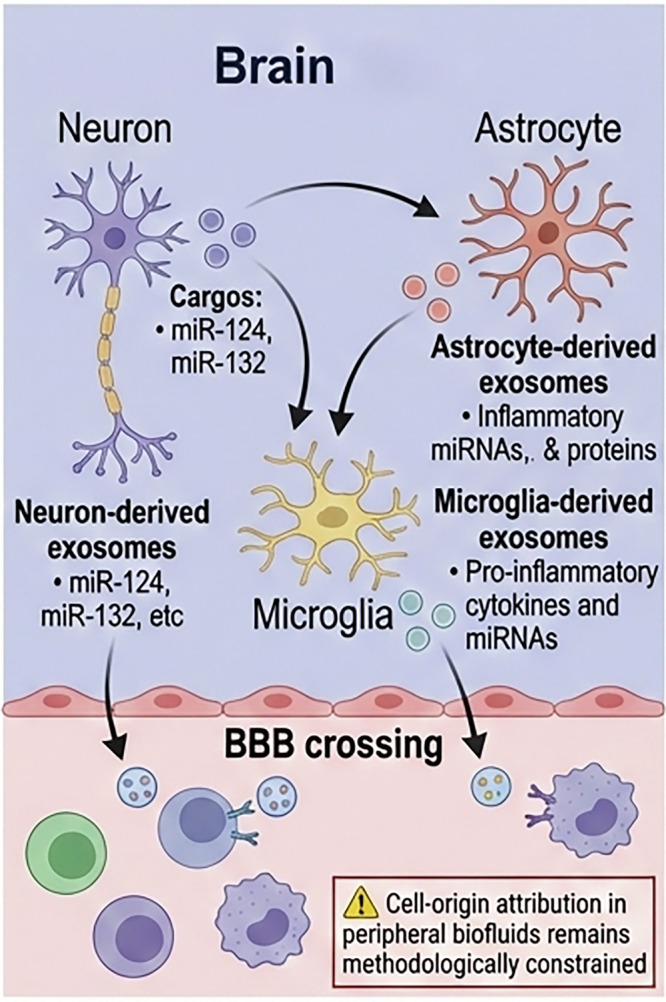
Cell type–specific exosomal signaling within the brain and implications for peripheral detection. Schematic illustration of exosome-mediated communication among major CNS cell populations, including neurons, astrocytes, and microglia. Neuron-derived exosomes are depicted as carrying regulatory miRNAs such as miR-124 and miR-132. Astrocyte-derived vesicles contain inflammatory-associated miRNAs and proteins. Microglia-derived exosomes are enriched in pro-inflammatory cytokines and immune-related miRNAs. Bidirectional vesicle exchange among these cell types highlights the dynamic integration of synaptic and immune signaling within neural circuits. The schematic further illustrates the potential trafficking of CNS-derived exosomes across the BBB into peripheral circulation. While such vesicle movement provides a conceptual basis for liquid biopsy–based biomarker development, accurate attribution of vesicle cellular origin in peripheral biofluids remains methodologically constrained.

Neuroinflammation represents a central biological dimension in a subset of individuals with depression. Circulating and CNS-derived exosomes from depressive cohorts have been reported to contain elevated levels of inflammatory mediators, including IL-6 and TNF-α [38–40]. Such vesicular cargo may reflect systemic immune activation and, under certain conditions, contribute to amplification of neuroimmune signaling. Microglia, the resident immune cells of the central nervous system, are highly responsive to extracellular vesicle-mediated communication. Vesicles enriched in pro-inflammatory cytokines, reactive oxygen species-associated molecules, or inflammatory miRNAs may influence microglial activation states, potentially altering synaptic integrity and neuronal viability (Fig. 2). Sustained neuroimmune activation has been associated with synaptic remodeling, dendritic spine loss, and network-level functional alterations that correlate with depressive symptom dimensions [70, 71]. However, the extent to which exosomes are causal drivers versus downstream reflections of inflammatory states remains unresolved.

Importantly, exosomes may also convey anti-inflammatory signals. MiRNAs such as miR-124 and miR-146a—both implicated in regulation of NF-κB-dependent pathways—have demonstrated immunomodulatory effects in experimental systems [72, 73]. MiR-146a primarily targets key inflammatory pathway molecules such as TNF receptor-associated factor 6 (TRAF6) and Interleukin-1 receptor-associated kinase 1 (IRAK1) to modulate the inflammatory response [74–78]. Vesicular delivery of these regulatory RNAs may attenuate excessive cytokine production and support restoration of neuroimmune equilibrium. Thus, exosomes appear to participate in bidirectional modulation of inflammatory tone, with functional outcome determined by cellular origin, microenvironmental context, and cargo composition.

Moreover, exosomes may influence central-peripheral immune communication through interactions with the BBB. Experimental studies indicate that vesicles can traverse or modulate BBB permeability under inflammatory conditions [79]. In depression—particularly in stress-related or inflammatory phenotypes—subtle BBB alterations have been reported (Fig. 2). Whether vesicle trafficking meaningfully contributes to peripheral-to-central immune signaling in humans remains an area of active investigation. Conversely, vesicles enriched in protective cargo have been hypothesized to support endothelial integrity, though direct evidence in depressive cohorts is limited.

This bidirectional regulatory mechanism highlights the pivotal role of exosomes in maintaining the dynamic balance of neuroinflammation. Exosomes can exacerbate the condition by promoting inflammation or mitigate it by delivering anti-inflammatory molecules, demonstrating unique complexity and diversity. Future research into the mechanisms of exosome-mediated neuroinflammation may not only elucidate the pathological mechanisms of depression but also provide critical clues for developing exosome-based innovative therapeutic strategies.

The brain-gut axis represents another systems-level pathway implicated in depression. This bidirectional network integrates central neural circuits, autonomic regulation, immune signaling, and gut microbiota–derived metabolites [12, 80–83]. Exosomes are increasingly recognized as potential mediators within this axis.

Microbiota-derived vesicles contain bioactive molecules—including miRNAs, proteins, and metabolites—that may influence host immune responses and, indirectly, central neural function [84]. Metabolites such as short-chain fatty acids (SCFAs) and tryptophan derivatives can modulate synaptic plasticity, inflammatory signaling, and stress responsivity [85]. Dysbiosis observed in subsets of depressed individuals may alter vesicular cargo profiles, potentially reshaping systemic inflammatory tone and neural signaling. For instance, miRNAs like miR-124 and miR-155, associated with anti-inflammatory and pro-inflammatory functions, respectively, are significantly altered in exosomes secreted by the gut microbiota of depression patients.

Conversely, central stress and inflammatory responses may influence gut barrier function and microbial composition through neuroendocrine and autonomic pathways, raising the possibility of reciprocal vesicle-mediated signaling [86–88]. Although compelling conceptually, direct mechanistic evidence delineating vesicle-specific contributions to brain-gut communication in human depression remains limited. Clarifying directionality, cargo specificity, and physiological relevance will be critical.

The bidirectional dynamics of the brain-gut axis suggest that exosomes may function not only as intermediaries of intersystem communication but also as potential modulators of this regulatory network. In depression, gut microbiota dysbiosis may reshape vesicular cargo profiles, altering inflammatory mediators, regulatory RNAs, and metabolic signals that influence central neuroimmune and synaptic processes. Reciprocally, stress-related and inflammatory changes within the CNS may affect gut barrier integrity and microbial composition through vesicle-associated signaling and neuroendocrine pathways, creating a self-reinforcing loop of peripheral and central dysregulation. Elucidating the origin, trafficking routes, and functional specificity of microbiota-associated exosomes—including their capacity to traverse or influence the BBB—will be essential to determine whether targeted modulation of microbial communities or vesicular cargo can meaningfully recalibrate brain-gut communication. Such mechanistic clarification may inform more precise, biologically grounded intervention strategies for depression.

Exosomes also intersect with neurotransmitter systems central to depression pathophysiology. Vesicular cargo has been reported to include enzymes involved in monoaminergic metabolism, such as MAO, which regulates dopamine, serotonin, and norepinephrine turnover [89]. MAO catalyzes neurotransmitter degradation, affecting their concentration in synaptic clefts, thereby regulating the strength and duration of synaptic signals. Additionally, exosomes may carry enzymes like glutamate dehydrogenase (GDH), which are involved in the metabolism of the excitatory neurotransmitter glutamate, helping balance excitatory and inhibitory signals [90]. Through modulation of metabolic pathways, vesicles may indirectly influence synaptic neurotransmitter availability.

Exosomes also transport proteins related to synaptic function, such as synaptic vesicle proteins (e.g., synaptophysin) and neurotransmitter transport molecules (e.g., serotonin transporter, SERT). These proteins influence neurotransmitter release mechanisms and reuptake efficiency, profoundly impacting synaptic signal transmission [91]. In depression, exosomal levels of these proteins may be altered, leading to neurotransmitter imbalances. For instance, elevated SERT levels in exosomes from depression patients may significantly reduce synaptic serotonin concentrations, exacerbating depressive symptoms [92].

Additionally, exosomes carry molecules that regulate synaptic plasticity, such as BDNF and its receptor tropomyosin receptor kinase B (TrkB) [93]. In depression patients, reduced BDNF levels in exosomes may be associated with weakened synaptic plasticity and neuronal dysfunction. However, whether vesicular transport actively shapes synaptic signaling or primarily mirrors intracellular changes remains to be determined. Furthermore, mitochondrial-related proteins identified within vesicles further suggest a potential link between metabolic regulation and synaptic efficiency. Given the emerging recognition of bioenergetic alterations in depression, vesicle-mediated redistribution of metabolic components warrants further investigation.

Taken together, exosomes may function as dynamic conduits linking immune activation, stress-related neuroendocrine signaling, gut–brain communication, and synaptic regulation. Rather than serving as singular pathogenic agents, they appear to reflect and potentially modulate the biological state of interconnected cellular networks. The relative contribution of vesicle-mediated signaling to depressive pathophysiology likely varies across inflammatory, metabolic, and stress-related subtypes of MDD. Establishing causal relationships will require longitudinal human studies, cell-type-specific tracing approaches, and mechanistic perturbation experiments. A systems-level understanding of exosome biology may ultimately refine current models of depression from isolated pathway disruption toward integrated network dysregulation.

## Evidence appraisal and methodological rigor

A critical appraisal of the current literature reveals that research investigating exosomes in depression broadly falls into three principal categories: preclinical mechanistic studies employing rodent stress paradigms, cross-sectional human biomarker investigations, and early-stage proof-of-concept therapeutic studies. Preclinical models—including chronic unpredictable mild stress (CUMS), chronic social defeat stress (CSDS), and related paradigms—have been instrumental in establishing biological plausibility, demonstrating that stress exposure alters exosomal cargo composition and that such vesicles can influence neuroinflammatory signaling, synaptic plasticity, and behavioral phenotypes [94–96]. However, these models capture only selected dimensions of depressive-like behavior and do not fully recapitulate the clinical heterogeneity, comorbidity burden, or chronicity observed in human major depressive disorder. Translational inference from rodent exosome dynamics to human pathophysiology must therefore be approached with caution.

Human studies, in contrast, predominantly adopt cross-sectional designs and frequently involve modest cohort sizes, often fewer than 100 participants. Diagnostic criteria, medication status, comorbid conditions, and inflammatory phenotypes are inconsistently characterized across studies, introducing substantial heterogeneity [97, 98]. Moreover, variability in pre-analytical and analytical procedures—including differences in biofluid selection (plasma versus serum), storage conditions, isolation methodologies (ultracentrifugation, size exclusion chromatography, polymer-based precipitation, or immunoaffinity capture), and downstream normalization strategies—limits reproducibility and cross-study comparability. Few investigations incorporate longitudinal sampling to determine whether exosomal signatures track symptom trajectories, treatment response, or relapse risk, thereby constraining conclusions regarding predictive validity.

Importantly, the evidentiary landscape remains largely associative rather than causal. Most human studies identify differential expression of specific exosomal microRNAs, proteins, or inflammatory mediators in patients compared with controls; however, such correlations do not establish mechanistic contribution. Only a limited subset of studies employ functional transfer paradigms—such as administration of patient-derived exosomes into rodent models—to probe causal effects on neurobehavioral outcomes [95, 99, 100]. Even in these cases, experimental conditions often do not fully disentangle whether observed behavioral changes arise from specific cargo molecules or from broader systemic differences between donor groups. As a result, causal inference remains provisional.

To advance the field toward mechanistic clarity and clinical translation, future investigations should prioritize longitudinal designs with repeated sampling across disease stages, standardized and consensus-driven isolation and characterization protocols aligned with international guidelines, multi-center validation cohorts to enhance reproducibility, and functional validation strategies extending beyond biomarker association. Integration of multi-omics profiling with experimental manipulation of candidate exosomal cargo will be essential to move from descriptive biomarker discovery to mechanistically grounded, therapeutically actionable insights.

## Exosomes as biomarkers for depression

Accumulating evidence suggests that extracellular vesicles, particularly exosomes, may provide a peripheral molecular window into biological processes associated with MDD. Alterations in vesicular cargo—including pro-inflammatory mediators, miRNAs, and proteins implicated in neurotransmitter regulation—have been reported across multiple biofluids in individuals with depression [99–101]. These observations support the hypothesis that exosomal signatures may reflect neuroinflammatory activation, synaptic dysregulation, and stress-related neuroendocrine alterations, although causality remains to be established [101–103].

Circulating exosomes isolated from plasma or serum are the most extensively studied source in depression research. Several reports describe elevated levels of inflammatory mediators, including IL-6 and TNF-α, within blood-derived vesicles from depressed cohorts [104, 105]. These findings are consistent with the broader literature linking systemic immune activation to subsets of MDD. Whether circulating exosomes actively propagate inflammatory signaling within the CNS or instead reflect peripheral immune status remains unresolved. Dysregulation of miRNA cargo is another recurrent finding. Reduced exosomal miR-16 has been associated with serotonergic signaling pathways, while miR-124—widely studied in microglial biology—may index neuroimmune state rather than direct neuronal injury. Importantly, most associations are correlational and derived from cross-sectional designs; replication in larger, longitudinal cohorts is required to clarify clinical utility (Table 1).

**Table 1:** Expanded candidate exosomal biomarkers in depression.

Biomarker	Biofluid	Evidence Type	Sample Size Range	Reproducibility
miR-124	Plasma (NDE-enriched)	Cross-sectional human	40–120	Moderate [95]
miR-132	Serum / Plasma	Human + rodent	30–100	Limited replication [172]
miR-16	Plasma / Serum	Human	50–150	Moderate [173]
miR-21	Plasma	Human + rodent (functional links to inflammation)	30–100	Limited [123]
miR-146a	Plasma	Human + rodent	30–90	Moderate [in inflammatory subgroups) [149]
miR-135a	Plasma	Human	40–100	Limited [174]
miR-34a	Plasma	Human + rodent	30–80	Limited [175]
miR-155	Plasma	Human	<100	Heterogeneous [100]
miR-9	Serum	Human	<80	Preliminary [176]
miR-29c	Plasma	Human	<100	Limited [177]
miR-223	Plasma	Human + inflammatory cohorts	40–120	Moderate (in immune-linked depression) [178]
IL-6 (protein)	Plasma exosomes	Human	<80	Heterogeneous [179]
TNF-related miRNAs	Plasma	Human	<100	Limited [180]
BDNF-related miRNAs (e.g., miR-30a, miR-206)	Plasma (TRD cohorts)	Human subgroup analysis	<60	Preliminary [181]
TSG101 (altered levels)	Plasma	Human	<80	Limited [116]
GFAP (post-TBI depression overlap)	Plasma (NDE)	Human (TBI cohorts)	<100	Preliminary [182]
Tau (phosphorylated)	Plasma (NDE)	Human (TBI-related depression risk)	<100	Preliminary [182]
CRP-associated cargo	Plasma	Human	<120	Heterogeneous [183]
Cortisol-regulated miRNAs	Plasma	Human	<80	Limited [184]
miR-181a	Plasma	Human + rodent stress models	40–100	Limited [185]

This table summarizes reported exosomal miRNAs and protein cargo identified in plasma- or serum-derived extracellular vesicles across human and translational studies of MDD, TRD, and TBI-associated depression. Evidence types include cross-sectional human cohorts, subgroup analyses, and combined human–rodent validation paradigms. Sample sizes are generally modest, and reproducibility varies across cohorts, reflecting heterogeneity in patient stratification, isolation workflows, and normalization strategies. Several candidates (e.g., miR-124, miR-16, miR-146a, and miR-223) demonstrate moderate reproducibility, particularly in inflammatory or immune-linked subgroups, whereas others remain preliminary or inconsistently replicated.

*NDE* neuron-derived exosome, *TRD* treatment-resistant depression, *TSG101* tumor susceptibility gene 101, *GFAP* glial fibrillary acidic protein, *Tau* microtubule-associated protein Tau, *CRP* C-reactive protein.

**Fig. 3:**
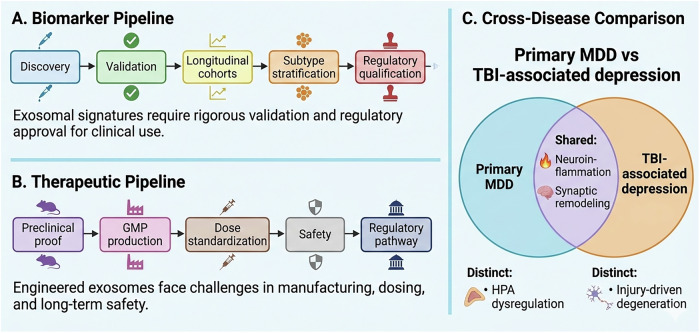
Translational framework and cross-disease comparison of exosomal signatures in depression. This schematic integrates biomarker development, therapeutic translation, and cross-disease profiling within a unified conceptual framework. **A** The biomarker pipeline encompasses discovery-phase profiling, multi-omics validation, longitudinal cohort studies, subtype stratification (e.g., inflammatory or treatment-resistant depression), and eventual regulatory qualification. **B** The therapeutic development pathway includes preclinical efficacy studies, dose standardization, Good Manufacturing Practice (GMP)-compliant production, safety evaluation, and regulatory classification within evolving biologics and advanced therapy medicinal product (ATMP) frameworks. **C** Comparative profiling between primary MDD and TBI-associated depression reveals overlapping mechanisms—such as chronic microglial activation, synaptic remodeling, and sustained neuroinflammation—alongside etiological distinctions, including stress-driven HPA axis dysregulation in primary MDD and injury-induced neurodegenerative processes in TBI-related depression.

Exosomes isolated from CSF provide closer biological proximity to CNS processes, albeit with practical limitations related to sample accessibility. Altered expression of enzymes involved in neurotransmitter metabolism, including MAO and GDH, has been reported in CSF-derived vesicles from individuals with depression [106]. These abnormalities likely contribute to neurotransmitter imbalances, impacting synaptic transmission and neural network activity. Furthermore, specific miRNAs, such as miR-9 and miR-132, associated with synaptic function and neuroplasticity, has also been described (Fig. 3).

Saliva represents a non-invasive and repeatable sampling matrix. Distinct miRNA profiles, including alterations in miR-21 and miR-146a, have been reported in salivary exosomes from depressed individuals [107, 108]. Given the involvement of these miRNAs in inflammatory signaling and gut-brain axis regulation, salivary vesicles may capture systemic components relevant to depressive phenotypes. However, standardization of collection and processing protocols is necessary before clinical implementation.

Across biofluids, exosomal cargo appears to reflect complementary biological dimensions—systemic immune activation, CNS-related signaling, and longitudinal monitoring potential. Analyzing exosomes from blood, CSF, and saliva provides data on systemic inflammation, CNS pathology, and non-invasive diagnostic potential, respectively. Advancing high-throughput technologies to characterize exosomal molecular features will help uncover depression-specific biomarkers. Nevertheless, current evidence primarily supports associative rather than mechanistically validated interpretations.

Exosomes possess several conceptual advantages as candidate biomarkers. Their presence in accessible biofluids enables repeated sampling, facilitating longitudinal assessment of disease trajectory and treatment response. Vesicular cargo integrates nucleic acids, proteins, lipids, and metabolites, potentially providing multidimensional molecular information. Exosomes can be extracted from blood, CSF, and saliva, with blood and saliva sampling particularly suitable for clinical use. Non-invasive sampling not only avoids discomfort for patients but also allows for repeated sampling to monitor disease progression dynamically [107, 109]. Salivary exosome analysis, due to its simplicity and repeatability, offers an ideal solution for populations such as children, the elderly, and those unable to undergo invasive procedures. Exosomes carry miRNAs, proteins, DNA, and metabolites that reflect the body’s physiological and pathological state. Studies have shown that depression-associated exosomal miRNAs (e.g., miR-16 and miR-124) and pro-inflammatory factors (e.g., IL-6 and TNF-α) directly correlate with disease severity and progression [102, 110, 111]. As dynamic monitoring tools, exosomes can provide real-time information for diagnosis, treatment efficacy assessment, and relapse prediction, enhancing comprehensive management of depression.

However, claims of high specificity should be interpreted cautiously. Surface markers such as CD63 and CD81 confirm vesicle identity but are not disease-specific. Similarly, while certain miRNA patterns may differentiate depressed individuals from controls in exploratory cohorts, reproducibility across platforms and populations remains limited [112, 113]. For instance, distinct miRNA expression patterns can not only differentiate depression patients from healthy individuals but also identify subtypes, offering scientific evidence for tailored therapeutic strategies. At present, exosome-based biomarkers should be considered investigational rather than clinically validated.

Future progress will depend on standardized isolation workflows, harmonized quantification metrics, and integration of multi-omics profiling with rigorous clinical phenotyping, ultimately advancing personalized and precision medicine in depression care.

## Cell-type resolution and biomarker validity

A central challenge in advancing exosome-based biomarkers for depression lies in accurately determining the CNS cellular origin of circulating vesicles. Many studies report alterations in so-called neuron-derived exosomes (NDEs) or astrocyte-derived exosomes (ADEs) isolated from peripheral biofluids using immunocapture strategies targeting surface markers such as L1 cell adhesion molecule (L1CAM) [114] or glutamate-aspartate transporter (GLAST) [115]. While these approaches represent important methodological innovations, the cellular specificity of these markers in plasma or serum remains a matter of ongoing debate. Emerging evidence indicates that L1CAM, long considered a neuronal adhesion molecule, may exist in soluble forms or be expressed beyond neurons under certain conditions, thereby complicating interpretation when used as a sole enrichment marker [116, 117]. Consequently, the assumption that L1CAM-positive vesicles in peripheral blood are exclusively neuron-derived may not be fully justified without additional validation.

Similar technical and conceptual challenges apply to the identification of microglia-derived exosomes. Microglia share multiple surface antigens and functional characteristics with peripheral monocytes and macrophages, particularly under inflammatory conditions. In systemic circulation, distinguishing CNS-resident microglial vesicles from those released by peripheral immune cells is therefore inherently difficult [70]. Given that depression is frequently accompanied by systemic immune activation, the risk of conflating central and peripheral immune-derived vesicles is nontrivial. This ambiguity is especially consequential when exosomal cargo is interpreted as reflecting brain-specific inflammatory or synaptic processes.

These limitations underscore a broader issue: assertions of cell-type-specific exosomal alterations in depression must be interpreted with methodological caution. Robust attribution of vesicle origin requires the use of multiple complementary marker panels rather than reliance on a single antigen, alongside orthogonal validation strategies integrating proteomic and transcriptomic profiling. Correlation with matched brain tissue data—whether derived from animal models or postmortem human samples—would further strengthen biological plausibility. Without such multi-layered validation, the translational validity of proposed diagnostic biomarkers remains provisional. Addressing the problem of cellular resolution is therefore not merely a technical refinement but a prerequisite for establishing reliable, brain-relevant exosome signatures capable of supporting precision psychiatry.

## Clinical heterogeneity in depression and exosomal signatures

MDD is increasingly recognized not as a unitary disease entity but as a biologically heterogeneous syndrome encompassing multiple pathophysiological trajectories. Symptom clusters, inflammatory burden, treatment responsiveness, metabolic status, and sex-specific factors all contribute to distinct clinical phenotypes that may arise from partially overlapping yet mechanistically divergent biological processes [118–122]. In this context, the interpretation of exosomal alterations in depression requires careful consideration of subtype stratification, as pooling heterogeneous patient populations may obscure biologically meaningful signal and limit translational applicability.

Emerging evidence suggests that exosomal cargo profiles may differ across clinically relevant subgroups. In individuals characterized by heightened systemic inflammation—often conceptualized as “inflammatory depression”—circulating exosomes have been reported to contain elevated levels of pro-inflammatory mediators, including IL-6–associated signaling components and TNF-regulated miRNAs [100, 123]. These findings align with the broader literature implicating immune dysregulation and microglial activation in a subset of patients. In contrast, treatment-resistant depression (TRD), a phenotype defined by insufficient response to conventional antidepressants, has been associated with alterations in neurotrophic signaling pathways, including differential expression of BDNF-related miRNAs within circulating vesicles [124, 125]. Such observations raise the possibility that exosomal markers may capture underlying biological mechanisms linked to pharmacoresistance, though validation remains limited.

Sex differences represent another underexplored dimension. Given the well-established disparity in depression prevalence and stress responsivity between males and females, sex-specific regulation of exosomal miRNAs—such as miR-124 and miR-132, both implicated in synaptic plasticity and neuroinflammatory signaling—may reflect hormonally modulated or epigenetically influenced pathways [126, 127]. However, few studies are adequately powered to interrogate sex-stratified effects, and most analyses adjust for sex as a covariate rather than examining it as a biological variable of interest. Similarly, depression occurring in the context of metabolic comorbidities—including obesity, insulin resistance, and metabolic syndrome—may involve gut-derived exosomal inflammatory mediators that contribute to brain–gut axis dysregulation. Altered intestinal permeability and systemic inflammation in such populations could shape circulating vesicle composition in ways that differ from non-metabolic depressive phenotypes [128].

Despite these preliminary observations, the majority of published studies do not stratify participants according to inflammatory status, treatment history, sex, or metabolic profile. As a result, subtype-specific exosomal signatures remain incompletely characterized, and precision-medicine implementation is constrained. Future biomarker development efforts must incorporate rigorous phenotypic characterization, predefined subgroup analyses, and adequately powered cohorts to determine whether exosomal cargo profiles can meaningfully distinguish biologically distinct forms of depression. Without deliberate integration of clinical heterogeneity into study design, the promise of exosome-based diagnostics risks being diluted by syndromic oversimplification.

## Therapeutic applications of exosomes in depression

Exosomes are being explored as candidate therapeutic platforms due to their biocompatibility, low immunogenicity, and ability to cross the blood–brain barrier. Most evidence derives from preclinical models. Recent comprehensive reviews have further advanced the field of exosome engineering and translational application [129–131], highlighting scalable manufacturing strategies, polymer-assisted stabilization approaches, and regulatory considerations relevant to psychiatric applications.

Exosomes can encapsulate small molecules, proteins, or nucleic acids and facilitate CNS delivery. This innovative strategy not only enhances the efficiency of drug delivery to the CNS but also opens new avenues for developing novel therapeutic approaches [132, 133]. Experimental studies suggest that exosome-mediated delivery of selective serotonin reuptake inhibitors (SSRIs) or neurotrophic factors may enhance brain targeting compared with conventional administration routes [134, 135]. Compared to traditional drug delivery methods, exosomal carriers significantly increase drug bioavailability, reducing nonspecific distribution in peripheral tissues, thereby minimizing adverse effects. Additionally, the membrane surface of exosomes can be genetically engineered to further enhance targeting to specific brain regions or cell types, thereby not only improving the therapeutic effect but also reducing drug dosages and lowering treatment costs (Fig. 3). However, quantitative comparisons of pharmacokinetics and clinical benefit remain limited.

Delivery of neurotrophic factors such as BDNF is theoretically attractive given its central role in synaptic plasticity and antidepressant response [136, 137]. Exosomal carriers may protect labile cargo from degradation and improve CNS bioavailability, though translational validation is pending.

Similarly, exosomal transfer of regulatory miRNAs—including miR-124 and miR-146a—has demonstrated anti-inflammatory effects in animal models [138–140]. Whether these effects translate to sustained behavioral improvement in humans remains uncertain These findings indicate that exosomal delivery of small RNAs offers a safe, efficient, and precise therapeutic strategy. Notably, exosomes can simultaneously carry multiple drugs or biomolecules, facilitating combination therapy. For example, exosome carriers can deliver both SSRIs and neuroprotective molecules, addressing neurotransmitter dysfunction while repairing neural damage. Moreover, exosomes can be designed to carry multiple miRNAs, enabling the modulation of neuroinflammation, synaptic function, and the gut-brain axis. This multi-targeted approach holds promise for significantly enhancing the overall therapeutic effect in depression.

Neuroplasticity, the ability of the nervous system to self-repair by altering its structure and function following injury or dysfunction, represents a critical therapeutic target in depression. In recent years, exosomes have attracted increasing attention for their role in modulating neuroplasticity, particularly in depression treatment [141, 142]. Exosomes can influence neuronal growth, development, and repair by carrying and transmitting neurotrophic factors, miRNAs, proteins, and other molecules, while also regulating neuronal interactions to support the reconstruction of neural networks.

Exosomal cargo, including neurotrophic factors such as BDNF and nerve growth factor (NGF), plays a pivotal role in promoting neuronal regeneration, repair, and growth. These factors activate specific signaling pathways to facilitate synapse formation and remodeling, thereby enhancing neuroplasticity. Additionally, exosomal miRNAs, such as miR-132, have been shown to exert significant neuroprotective effects. miR-132 regulates the expression of genes associated with neuronal growth and synaptic plasticity, enhancing dendritic spine formation, improving synaptic function, and promoting more efficient information transmission between neurons [143, 144].

Beyond protecting individual neurons, exosomes also play a crucial role in maintaining the stability of neural networks and preserving synaptic connections. In depression, functional impairments in synaptic connections and neural network stability lead to significant alterations in mood regulation, cognition, and behavior [102, 145]. Exosomes, by transporting specific molecules, aid in the repair and protection of these damaged synaptic connections, facilitating the reconstruction of neural networks, thereby improving cognitive function and emotional states. Research has shown that exosomes not only directly promote synapse formation but also improve overall neural function by modulating immune responses and reducing neuroinflammation. Nevertheless, mechanistic dissection is incomplete, and functional specificity within defined brain circuits remains to be clarified.

Stem cell-derived exosomes, endowed with a rich cargo of bioactive molecules, are emerging as a significant tool in the treatment of depression. Due to exosomes’ diverse origins, stem cell-derived exosomes have shown promising potential in treating various neurological diseases, particularly in depression, a complex affective disorder, exhibiting unprecedented effects.

Mesenchymal stem cell (MSC)-derived exosomes represent the most extensively investigated subclass of stem cell-derived extracellular vesicles in neuropsychiatric research. MSCs are characterized by multipotent differentiation capacity and broad immunomodulatory properties, mediated in part through paracrine signaling. Their secreted vesicles encapsulate bioactive molecules that may influence immune regulation, neuronal survival, and tissue remodeling [146]. In rodent models of chronic stress and depression-like behavior, administration of MSC-derived exosomes has been associated with partial normalization of behavioral phenotypes, including anxiety-like behavior, social withdrawal, and reduced locomotor activity [147, 148]. Although these findings are promising, they derive primarily from preclinical paradigms and require cautious interpretation. The mechanisms underlying this effect are primarily reflected in the following aspects:

### Modulation of neuroinflammation

Chronic low-grade neuroinflammation is implicated in a subset of individuals with major depressive disorder. MSC-derived exosomes have been shown in experimental systems to carry regulatory microRNAs, including miR-21 and miR-146a, that modulate inflammatory signaling pathways [149–151]. miR-21 has been linked to regulation of immune-related gene expression and attenuation of glial activation, whereas miR-146a targets components of the NF-κB pathway, including TRAF6 and IRAK1. Through these mechanisms, vesicle-associated cargo may shift microglial activation states and reduce pro-inflammatory cytokine production [149–151]. Whether these effects translate into durable clinical benefit in humans remains to be determined.

### Neurotrophic and neuroprotective signaling

MSC-derived vesicles are enriched in neurotrophic factors, including BDNF and NGF [150, 152]. BDNF plays a central role in synaptic plasticity, neuronal survival, and stress resilience. Delivery of neurotrophic cargo via exosomes has been associated with restoration of synaptic protein expression and dendritic morphology in animal models. However, it remains unclear whether vesicular transfer directly drives these changes or reflects broader paracrine effects of MSC signaling.

### Structural and metabolic support

Chronic stress paradigms are associated with structural alterations in the hippocampus and prefrontal cortex. Experimental data suggest that MSC-derived exosomes may influence markers of neuronal apoptosis, synaptic density, and metabolic function [150]. These findings raise the possibility that vesicles contribute to neural network stabilization and tissue remodeling under stress conditions, though mechanistic specificity and cell-type targeting remain incompletely characterized.

### Safety and translational considerations

Compared with direct stem cell transplantation, exosome-based approaches may offer theoretical safety advantages, including lower immunogenicity and absence of proliferative risk. Vesicles are biodegradable and capable of crossing the BBB under certain conditions [153–156]. Nonetheless, clinical translation requires rigorous standardization of manufacturing, dosing metrics, biodistribution profiling, and long-term safety evaluation.

In summary, stem cell-derived exosomes exhibit immense potential in the treatment of depression. Through the synergistic effects of multiple mechanisms, MSC-derived exosomes may influence depressive phenotypes through coordinated modulation of neuroinflammatory signaling, neurotrophic support, and structural plasticity. However, current evidence is largely preclinical, and definitive causal pathways have not been established. Establishing reproducible potency assays, clarifying cargo-function relationships, and conducting controlled human studies will be essential before MSC-derived vesicles can be considered viable therapeutic candidates in depression.

Recent studies have begun to explore exosomes derived from induced pluripotent stem cells (iPSCs) and their derivatives as a potential source for regenerative and immunomodulatory vesicles [153, 154]. Preclinical work has shown that iPSC-derived exosomes can attenuate microglial inflammatory responses by delivering noncoding RNA cargo in vitro, suggesting a possible mechanism for modulating neuroinflammatory pathways relevant to neuropsychiatric conditions [157]. Importantly, iPSCs offer a theoretically inexhaustible and more standardizable source of vesicles compared with somatic stem cells, which may be advantageous for scalable therapeutic development. However, applications targeting depressive phenotypes remain at an early stage of investigation, with no direct evidence in validated behavioral models or clinical settings to date; thus, any therapeutic potential should be presented with appropriate caveats and positioned as an emerging area for future research.

The multiple roles of exosomes in depression therapy reveal their potential as a novel therapeutic tool. From drug delivery carriers to neuroplasticity modulation, and stem cell-derived exosome applications, exosomes are reshaping the research landscape in depression therapy. In the future, further optimization of exosome production, loading, and delivery technologies, along with deeper exploration of their mechanisms of action, will likely position exosomes as a key component in personalized treatment strategies, providing more efficient and precise therapeutic options for patients.

## Exosomes in depression secondary to traumatic brain injury and neurological comorbidities

Depression frequently arises not only as a primary psychiatric disorder but also as a neuropsychiatric sequela of neurological injury and disease, most notably traumatic brain injury (TBI). The prevalence of post-TBI depression underscores the necessity of situating exosome research within a broader cross-disease framework. TBI induces a cascade of pathophysiological processes—including chronic microglial activation, BBB disruption, synaptic remodeling, axonal injury, and persistent neuroinflammation—that substantially overlap with mechanisms implicated in major depressive disorder [158, 159]. These shared biological substrates raise the possibility that exosomal signaling may represent a convergent molecular interface linking injury-induced and stress-related depressive phenotypes.

Exosomes are now recognized as central mediators in TBI pathology. Following mechanical injury, CNS-resident cells release vesicles enriched in inflammatory mediators, cytoskeletal proteins, and injury-associated molecules such as tau and glial fibrillary acidic protein (GFAP). These vesicles can propagate inflammatory signaling across neural networks, modulate microglial and astrocytic activation states, and potentially contribute to the spread of neurodegenerative processes. Notably, neuron-derived exosomes isolated from individuals after TBI have demonstrated alterations in cargo composition—particularly in proteins and miRNAs related to synaptic plasticity, neuroinflammation, and stress signaling—that resemble patterns reported in primary depression [160, 161]. Such observations suggest the existence of partially shared molecular pathways underpinning depressive symptomatology across etiologically distinct conditions.

Despite these convergences, important mechanistic distinctions likely persist. In primary MDD, exosomal alterations are frequently conceptualized within the framework of chronic stress exposure, hypothalamic-pituitary-adrenal (HPA) axis dysregulation, glucocorticoid signaling imbalance, and psychosocial vulnerability. By contrast, in TBI-associated depression, exosomal cargo may more directly reflect injury-driven neurodegeneration, structural network disruption, and sustained neuroimmune activation initiated by mechanical insult. The temporal dynamics may also differ: whereas primary depression often evolves gradually in the context of chronic stress, TBI-related exosomal changes may follow an acute injury phase followed by prolonged secondary inflammatory processes. These distinctions have implications for biomarker specificity and therapeutic targeting (Fig. 3).

Beyond TBI, similar considerations extend to other neurological comorbidities in which depression is highly prevalent, including stroke, neurodegenerative disorders, and epilepsy. In each case, exosome-mediated intercellular communication may integrate injury signals with mood-regulatory circuitry dysfunction [59, 162, 163]. However, systematic comparative analyses of exosomal cargo profiles across primary depression and secondary depression arising from neurological conditions remain scarce. Direct cross-cohort profiling, harmonized isolation methodologies, and longitudinal designs are needed to delineate shared versus disorder-specific vesicular signatures. Such comparative approaches would not only refine biomarker interpretation but also facilitate the identification of common therapeutic nodes within a broader neuropsychiatric network model.

Integrating TBI and other neurological comorbidities into the conceptual framework of exosome research thus expands the translational relevance of the field. Rather than viewing depression in isolation, positioning exosomal signaling within a cross-disease continuum may reveal both convergent inflammatory and synaptic pathways and distinct etiological signatures. This strategy holds promise for advancing biomarker discovery, improving diagnostic precision, and informing targeted interventions across neuropsychiatric conditions characterized by overlapping biological vulnerability.

## Therapeutic translation, methodological standardization, and future directions

Although engineered and stem cell-derived exosomes have demonstrated compelling therapeutic effects in preclinical models of depression—attenuating neuroinflammation, restoring synaptic plasticity, and modulating stress-responsive signaling pathways—their progression toward clinical application remains constrained by substantial scientific, technical, and regulatory challenges. The conceptual appeal of exosome-based interventions lies in their capacity to cross the blood–brain barrier, deliver bioactive cargo with relative specificity, and recapitulate endogenous intercellular communication mechanisms. However, enthusiasm for their therapeutic promise must be tempered by a realistic appraisal of translational readiness.

A foundational barrier concerns the lack of methodological standardization in exosome isolation, characterization, and potency assessment. Exosomes are heterogeneous nanoscale vesicles enriched with miRNAs, proteins, lipids, and other bioactive molecules; consequently, the purity and integrity of isolated preparations directly influence both mechanistic interpretation and therapeutic efficacy. Widely used isolation techniques—including ultracentrifugation, size-exclusion chromatography (SEC), polymer-based precipitation, immunocapture strategies targeting canonical markers such as CD63 or CD81, and emerging microfluidic platforms—each possess inherent advantages and limitations [164–166]. Importantly, no universally accepted gold standard currently exists for exosome isolation, and methodological choice often reflects laboratory preference rather than consensus-driven validation. Although adherence to the Minimal Information for Studies of Extracellular Vesicles (MISEV) guidelines proposed by the International Society for Extracellular Vesicles (ISEV) has improved reporting transparency and experimental rigor, compliance remains variable, and substantial heterogeneity persists in pre-analytical handling, purification workflows, and characterization criteria.

Ultracentrifugation, while historically dominant, is labor-intensive and susceptible to protein or lipid contamination. SEC offers improved purity and suitability for high-throughput applications but may introduce variability related to column conditions and flow dynamics. Immunocapture techniques provide subtype selectivity but depend on antibody specificity, are relatively costly, and risk bias if marker expression shifts under pathological conditions. Microfluidic approaches promise rapid and low-volume processing yet remain limited by accessibility and scalability. The absence of harmonized protocols across laboratories generates variability in yield, purity, and cargo composition, complicating cross-study comparisons and undermining reproducibility—particularly in the context of clinical translation (Table 2). Standardization of isolation workflows therefore remains a prerequisite for cross-cohort reproducibility and eventual regulatory approval [165].

**Table 2:** Comparison of commonly used and emerging exosome isolation and enrichment methods.

Method	Advantages	Limitations	Clinical Suitability
Differential Ultracentrifugation (UC)	Gold standard in basic research; no chemical additives; scalable for large volumes	Labor-intensive; low specificity; co-isolation of protein aggregates and lipoproteins; batch variability	Research use; limited direct clinical translation [164]
Density Gradient Ultracentrifugation	Higher purity than conventional UC; improved separation by buoyant density	Time-consuming; technically demanding; low throughput	Advanced research; potential reference method [186]
Size Exclusion Chromatography (SEC)	Improved purity; gentle on vesicles; reproducible; suitable for plasma	Requires optimization; column variability; moderate throughput	Moderate; promising for clinical-grade workflows [187]
Polymer-Based Precipitation (e.g., PEG kits)	Simple; high yield; compatible with small sample volumes	Low purity; protein contamination; variable reproducibility	Exploratory studies only; not ideal for clinical use [165]
Immunoaffinity Capture (CD63, CD81, CD9)	Enrichment of specific vesicle populations; high specificity for defined markers	Expensive; dependent on antibody specificity; potential bias in subpopulation capture	Biomarker discovery; limited therapeutic-scale use [188]
Cell-Type–Specific Immunocapture (e.g., L1CAM, GLAST)	Enables putative neuron- or astrocyte-derived exosome enrichment	Marker specificity debated; possible peripheral contamination	Research only; requires orthogonal validation [117]
Magnetic Bead–Based Immunocapture	Improved efficiency; compatible with small volumes; semi-automatable	Antibody cost; marker dependency; risk of nonspecific binding	Translational biomarker research [189]
Ultrafiltration (Membrane-based)	Rapid; scalable; cost-effective	Membrane clogging; vesicle deformation risk; moderate purity	Adjunct method; not standalone clinical standard [190]
Tangential Flow Filtration (TFF)	Scalable; GMP-compatible; suitable for large-volume production	Requires specialized equipment; optimization needed	High suitability for therapeutic manufacturing [168]
Microfluidic-Based Isolation	Low sample volume; rapid processing; integration with on-chip analysis	High cost; device variability; limited large-scale validation	Emerging; potential for point-of-care diagnostics [191]
Acoustic/Dielectrophoretic Separation	Label-free; preserves vesicle integrity	Early-stage technology; limited availability	Experimental [192]
Asymmetric Flow Field-Flow Fractionation (AF4)	High-resolution size-based separation; research-grade purity	Complex instrumentation; low throughput	Advanced research applications [193]
Anion Exchange Chromatography	Scalable; compatible with GMP; improved purity control	May co-isolate similarly charged particles	Therapeutic development (emerging) [194]
Combined Approaches (e.g., UC + SEC)	Improved purity and specificity; flexible workflows	Increased processing time; higher cost	Translational research; potential clinical standard [195]

This table summarizes the principal methodologies employed for extracellular vesicle isolation, outlining their respective advantages, technical limitations, and relative suitability for clinical translation. Approaches range from conventional differential ultracentrifugation and density gradient centrifugation to chromatography-based techniques, immunoaffinity capture strategies, membrane filtration systems, and emerging microfluidic and physicochemical separation technologies. While ultracentrifugation remains widely used in basic research, alternative platforms such as size exclusion chromatography (SEC), tangential flow filtration (TFF), and anion exchange chromatography offer improved scalability and compatibility with GMP requirements. Immunoaffinity-based methods enable enrichment of defined vesicle subpopulations but are constrained by marker specificity and cost considerations. Emerging label-free and microfluidic technologies provide rapid and low-volume processing but currently lack large-scale validation.

*UC* ultracentrifugation, *SEC* size exclusion chromatography, *PEG* polyethylene glycol, *TFF* tangential flow filtration, *AF4* asymmetric flow field-flow fractionation.

Quality control and characterization further require multimodal validation. Transmission electron microscopy remains indispensable for morphological confirmation but may introduce preparation artifacts. Nanoparticle tracking analysis enables quantification of particle size distribution and concentration, though it may be influenced by co-isolated particles. Western blotting confirms canonical marker expression yet lacks particle-level resolution. Advances in high-sensitivity flow cytometry now permit single-vesicle analysis, albeit with technical constraints related to size detection thresholds. Complementary high-throughput omics approaches—including next-generation sequencing for RNA profiling and mass spectrometry for proteomic and lipidomic characterization—are increasingly essential for defining exosomal molecular signatures [167]. The integration of these orthogonal techniques is critical for establishing reproducible standards that support both mechanistic discovery and therapeutic development.

Dosing standardization presents an additional conceptual challenge. Unlike small-molecule drugs defined by precise molar concentrations, exosome preparations may be quantified by particle number, total protein content, RNA yield, or surface marker abundance, with no consensus regarding the most biologically meaningful metric. Variability in isolation efficiency and cargo loading further complicates interpretation of dose–response relationships [167]. Without validated potency assays and standardized reporting metrics, extrapolation from preclinical dosing paradigms to human clinical trials remains uncertain.

Scalability and manufacturing consistency under Good Manufacturing Practice (GMP) conditions introduce further complexity. Clinical deployment requires rigorous quality control, reproducible batch production, validated purification pipelines, and careful monitoring of donor cell stability and cargo fidelity during expansion, storage, and transport [168]. Achieving robust batch-to-batch consistency while preserving functional activity is technically demanding and necessitates coordinated methodological standardization and regulatory oversight.

Safety considerations are equally paramount. Although exosomes are often characterized as inherently biocompatible, their long-term biodistribution, tissue persistence, and potential off-target effects—particularly following repeated systemic administration—remain incompletely defined. Risks of unintended immune activation, accumulation in non-target organs, or theoretical oncogenic effects associated with proliferative signaling cargo warrant systematic preclinical evaluation [169]. Engineered vesicles incorporating nucleic acids or gene-editing components introduce additional biosafety considerations that require comprehensive toxicological assessment.

Compounding these scientific challenges is an evolving regulatory landscape. Exosome-based therapeutics occupy a transitional space between biologics, cell-derived products, and advanced therapy medicinal products (ATMPs). Regulatory classification influences manufacturing requirements, trial design, pharmacovigilance obligations, and commercialization pathways [170]. Given the heterogeneity of exosome sources and engineering strategies, early and proactive engagement with regulatory authorities is essential to define product characterization criteria and approval frameworks.

Beyond therapeutic deployment, another critical frontier lies in validating exosome-specific biological actions. Exosomes are secreted by nearly all cell types, and multiple populations coexist within biological fluids. Accurately attributing functional effects to vesicles derived from specific cellular origins—particularly within the CNS—remains technically demanding. Surface markers used for enrichment may not be exclusively cell-specific, and their expression may vary across physiological and pathological states [171]. In vivo tracking of exosomes is further complicated by their nanoscale dimensions, dynamic biodistribution, and overlapping physical characteristics. Emerging labeling strategies, including fluorescent dyes, nanoparticle conjugation, and bioluminescent reporters, enable experimental tracing in preclinical models; however, their clinical translation is constrained by concerns regarding stability, signal specificity, and biocompatibility. Advanced imaging modalities, super-resolution microscopy, and mass spectrometry imaging are beginning to refine spatial localization, yet precise mapping of vesicle origin and target engagement in human tissues remains an unresolved challenge.

Equally important is elucidating the functional specificity of exosomal cargo. MicroRNAs such as miR-21, miR-146a, miR-16, and miR-124, as well as protein and lipid mediators, have been implicated in neuroinflammatory regulation, neuronal survival, and synaptic remodeling. However, the regional specificity of their actions within distinct brain circuits, the influence of cellular origin on functional outcome, and the extent to which cargo alterations reflect causal mechanisms versus downstream epiphenomena remain incompletely understood. Systematic integration of multi-omics profiling with functional perturbation studies will be essential to disentangle these relationships.

Looking forward, high-throughput sequencing, quantitative proteomics, metabolomics, and integrative systems biology approaches offer powerful tools to systematically define exosomal molecular landscapes in depression. Large-scale, well-phenotyped cohorts incorporating longitudinal sampling and subtype stratification will be necessary to identify robust, clinically meaningful biomarkers. In parallel, advances in bioengineering may enable the development of exosome-based precision delivery platforms tailored to individual inflammatory, neurotrophic, or metabolic profiles. Engineered vesicles designed to modulate specific pathological pathways hold theoretical promise for personalized intervention strategies. However, realization of this vision will require rigorous validation, standardized methodologies, and careful alignment with regulatory frameworks.

Taken together, while exosome research represents a promising frontier in neuropsychiatric science, its translation into reliable diagnostic tools and safe, effective therapeutics remains at an early developmental stage. Progress will depend not only on biological innovation but also on methodological harmonization, mechanistic clarity, and responsible regulatory engagement. A balanced integration of enthusiasm and rigor is essential to ensure that the field advances on a scientifically robust and clinically meaningful trajectory.

## Conclusion

Exosomes have emerged as central mediators of intercellular communication within the neuroimmune and neuroendocrine networks implicated in depression, positioning them at the intersection of pathogenesis, biomarker discovery, and therapeutic innovation. Accumulating evidence indicates that exosomal cargo—including miRNAs, proteins, lipids, and metabolites—participates in the regulation of neuroinflammation, synaptic remodeling, hypothalamic-pituitary-adrenal axis dynamics, and brain-gut axis signaling. These observations provide a conceptual framework in which vesicle-mediated communication contributes to the integration of peripheral and central processes underlying depressive phenotypes. At the same time, careful appraisal of the literature underscores that much of the current evidence remains associative, with causal mechanisms and cell-of-origin specificity requiring further validation through longitudinal, multi-center, and functionally grounded investigations.

From a diagnostic perspective, circulating exosomes offer a compelling, minimally invasive window into brain-relevant molecular processes. Candidate cargo molecules—such as miR-16, miR-124, and inflammation-related microRNAs—have shown potential as biomarkers capable of reflecting disease state, inflammatory subtype, or treatment responsiveness. However, methodological heterogeneity in isolation protocols, variability in characterization techniques, and limited cohort stratification constrain reproducibility and clinical interpretability. Establishing standardized pipelines and integrating multi-omics profiling with rigorous phenotypic characterization will be essential to transform exploratory signatures into clinically actionable tools.

Therapeutically, exosomes represent a promising but still early-stage platform. Preclinical studies suggest that native or engineered vesicles can modulate neuroinflammatory cascades, enhance neuroplasticity, and serve as delivery vehicles for targeted molecular interventions. Yet substantial translational challenges persist, including dose standardization, scalable GMP-compliant manufacturing, long-term safety evaluation, and evolving regulatory classification. Moreover, precise attribution of cell-specific origin and functional cargo effects remains technically demanding, particularly in complex neuropsychiatric contexts and in secondary depression associated with neurological injury.

Looking forward, progress in the field will depend on the convergence of methodological rigor, mechanistic clarity, and translational realism. High-throughput sequencing, quantitative proteomics, advanced imaging, and systems-level integration should be coupled with longitudinal study designs and subtype-specific analyses to refine biological precision. Comparative investigations across primary and comorbidity-associated depression, such as that arising after traumatic brain injury, may further delineate shared and disorder-specific vesicular pathways. Through interdisciplinary collaboration spanning neuroscience, psychiatry, bioengineering, and regulatory science, exosome research has the potential to evolve from descriptive association toward mechanistically informed diagnostics and responsibly developed therapeutics. With sustained rigor and innovation, exosomes may ultimately contribute to a more precise and biologically grounded paradigm for understanding and treating depression.
